# Legal Notes for Institutional Workers

**Published:** 1916-06-03

**Authors:** 


### LEGAL NOTES FOR INSTITUTIONAL WORKERS.

### Liability of Hospital and School Managers Compared.

The valuable principle laid down in *Hillyer* v. *Governors of St. Bartholomew's Hospital*, and in *Foote* v. *Directors of Greenock Infirmary* to the effect that the managers of a hospital are not responsible for the acts of the medical staff was recently sought to be applied to a School Board, in one of whose schools a pupil had been injured in the course of an experiment in chemistry, and against whom the guardians of the boy brought an action founding on the alleged fault of the teacher, who was the Board's servant. The defendants argued that the position of a teacher was analogous to that of a medical man, and that just as it had been held that hospital directors could not be expected to control a surgeon in his specially skilled work but had discharged their duty when they had appointed a competent and duly qualified man to the work, so a popularly elected body like an Education Authority should not be asked to accept responsibility for a science master, but should be deemed clear of all liability once they had appointed a properly certificated and well-recommended man. The Court declined to entertain that argument, following the *dicta* of Fletcher Moulton, L.J., in *Smith* v. *Martin*, (1911) 2 K.B. 775, who there said: "The position of a medical man is a very special one, and bears no resemblance to that of a teacher in an elementary school. The layman is presumably incapable of judging the right treatment to be adopted by the medical man, and accordingly he is not required to interfere with it, or even warranted in doing so. But the Education Authority can and ought to take cognisance of the way in which teaching is given; and, although it is rightly guided to a great extent by the advice of the teacher, it can direct the teacher what to do and what not to do, and it is clear he must obey."

### Fees of Doctors as Witnesses.

The same statute more than any other necessitates medical men appearing and giving evidence. According to practice, if such skilled witnesses make investigations or examinations prior to trial to qualify them to give evidence at the trial, they are, as a general rule, allowed special charges for such work in addition to their fees as witnesses in the case. In a recent case a doctor had done preliminary work in view of a trial, but an arrangement was afterwards arrived at and he did not require to appear. A motion to allow special charges to the skilled medical witness was objected to as incompetent on the ground that he had not appeared as a witness in the case; but the Court granted the motion, the judge remarking, "otherwise the person so employed might be deprived of suitable recompense for work done."

